# Human surveillance and phylogeny of highly pathogenic avian influenza A(H5N8) during an outbreak in poultry in South Africa, 2017

**DOI:** 10.1111/irv.12724

**Published:** 2020-02-14

**Authors:** Ziyaad Valley‐Omar, Alicia Cloete, Reneé Pieterse, Sibongile Walaza, Yusrah Salie‐Bassier, Mikhail Smith, Nevashan Govender, Mpho Seleka, Orienka Hellferscee, Phillip Senzo Mtshali, Mushal Allam, Arshad Ismail, Tasneem Anthony, Michelle Seutloali, Kerrigan McCarthy, Lesley van Helden, Cheryl Cohen, Florette Kathleen Treurnicht

**Affiliations:** ^1^ National Institute for Communicable Diseases of the National Health Laboratory Service Johannesburg South Africa; ^2^ Department of Pathology Division of Medical Virology University of Cape Town South Africa; ^3^ Department of Agriculture Western Cape Provincial Veterinary Laboratory Stellenbosch South Africa; ^4^ Veterinary Services Western Cape Department of Agriculture South Africa; ^5^ School of Public Health Faculty of Health Sciences University of the Witwatersrand Johannesburg South Africa; ^6^ School of Pathology Faculty of Health Sciences University of the Witwatersrand Johannesburg South Africa

**Keywords:** avian influenza, H5N8, South Africa

## Abstract

**Background:**

In June 2017, an outbreak of the highly pathogenic avian influenza A(H5N8) was detected in commercial poultry farms in South Africa, which rapidly spread to all nine South African provinces.

**Objectives:**

We conducted active surveillance for the transmission of influenza A(H5N8) to humans working with infected birds during the South African outbreak.

**Methods:**

Influenza A(H5N8)‐positive veterinary specimens were used to evaluate the ability of real‐time PCR‐based assays to detect contemporary avian influenza A(H5N8) strains. Whole genome sequences were generated from these specimens by next‐generation sequencing for phylogenetic characterization and screening for mammalian‐adaptive mutations.

**Results:**

Human respiratory samples from 74 individuals meeting our case definition, all tested negative for avian influenza A(H5) by real‐time PCR, but 2 (3%) were positive for human influenza A(H3N2). 54% (40/74) reported wearing personal protective equipment including overalls, boots, gloves, masks, and goggles. 94% (59/63) of veterinary specimens positive for H5N8 were detected on an influenza A(H5) assay for human diagnostics. A commercial H5N8 assay detected H5 in only 6% (3/48) and N8 in 92% (44/48). Thirteen (13/25; 52%) A(H5N8) genomes generated from veterinary specimens clustered in a single monophyletic clade. These sequences contained the NS (P42S) and PB2 (L89V) mutations noted as markers of mammalian adaptation.

**Conclusions:**

Diagnostic assays were able to detect and characterize influenza A(H5N8) viruses, but poor performance is reported for a commercial assay. Absence of influenza A(H5N8) in humans with occupational exposure and no clear impression of molecular adaptation for mammalian infection suggest that this avian pathogen continues to be low‐risk human pathogen.

## 1 INTRODUCTION

In June 2017, an outbreak of the highly pathogenic avian influenza (HPAI), A(H5N8) was detected on commercial poultry farms in the Mpumalanga Province of South Africa and reported to the World Organization for Animal Health (OIE).^1^ This followed soon after reported outbreaks in neighboring Zimbabwe.^2^ Over the months that followed, the virus rapidly spread across the country to all nine South African provinces resulting in the death and culling of millions of commercially farmed birds as well as mortalities in several species of wild birds.^1^

People that are in close contact with infected birds or carcasses are regarded as being at potentially elevated risk of acquiring avian influenza (AI) as the virus may be transmitted through infectious secretions and excretions.^3^, ^4^, ^5^, ^6^ While thousands of people worldwide working in close contact with infected birds have been exposed to influenza A(H5N8), no human infections have been reported to date.^6^, ^7^, ^8^ This suggests the risk of human infection is low. However, the risk in South Africa may differ when compared to other countries as a result of an HIV prevalence of 18.8% within the 15‐49 year old population.^9^ It should be noted that influenza A(H5N8) has been shown to infect and be mildly pathogenic in ferrets and mice.^5^, ^10^, ^11^ Mutations facilitating adaptation of avian influenza viruses for the infection of mammalian hosts consist of individual or limited sets of point mutations in specific gene segments like PB2 (L89V, E627K, D701N) and hemagglutinin (HA) (A149V) and NS1 (P42S).^11^, ^12^, ^13^, ^14^, ^15^, ^16^, ^17^, ^18^, ^19^, ^20^, ^21^, ^22^ Thus, while influenza A(H5N8) poses limited zoonotic transmission risk, given its evolutionary history and relatively simple genetic adaptations required for potential mammalian infection, it could pose a potential pandemic risk.

The one health concept recognizes that human, animal, and environmental health is interlinked and encourages close collaboration between respective health authorities to work toward achieving health for all.^23^ Close interaction and possible collaboration between human and animal health authorities during influenza surveillance plays an important role in ensuring that both sectors are aware and prepared to detect, respond, and control potential zoonotic influenza viruses. In this study, we conducted active surveillance for the transmission of influenza A(H5N8) to humans working with infected birds during the 2017 outbreak in South Africa. We also evaluated the ability of 2 real‐time PCR‐based assays to detect avian influenza A(H5N8) strains that circulated in birds during the outbreak period and characterized them by genome sequencing for known adaptive mutations that could augment host range and virulence.

## 2 METHODS

### 2.1 Human surveillance for potential zoonotic transmission

The Outbreak Response Unit (ORU) and Centre for Respiratory Diseases and Meningitis (CRDM) of the National Institute for Communicable Diseases (NICD) launched active epidemiological and laboratory investigations to screen in‐contact workers and animal health personnel for influenza A(H5N8) viruses. A case under investigation was defined as a person who presented with any one or combination of symptoms including cough, fever, sore throat, runny nose, difficulty breathing, or conjunctivitis while also having a documented history of exposure (direct contact or proximity of <15 m) to potentially infected birds (alive or dead) or having worked in a poultry house with potentially infected birds, in the 10 days preceding the onset of symptoms. Active surveillance was conducted on three affected commercial poultry farms located in Mpumalanga and Gauteng Provinces. Demographic and clinical data, as well as information on personal protective equipment use and hand hygiene practices, were collected by an interviewer using a case investigation form (CIF). Oropharyngeal or combined oropharyngeal and nasal swabs were placed in Universal Transport Medium (UTM) (Copan Italia, Brescia, Italy) and were transported to the NICD within 24 hours of collection for testing.

In addition, passive surveillance included samples from animal health personnel meeting the same case definition and who were involved in outbreak response activities on multiple A(H5N8)‐affected farms in the Western Cape Province (WCP) and from workers from a bird park with laboratory‐confirmed A(H5N8)‐positive birds in Gauteng Province were submitted to the CRDM laboratory after completion of the CIF. Specimens from patients meeting the case‐under‐investigation definition referred from private and state pathology laboratories were also included.

### 2.2 Personal protective equipment (PPE) recommendations

The NICD recommended that all people that work in close contact with poultry should wear appropriate PPE when handling potentially infected birds, carcasses, contaminated material, or when cleaning equipment and production houses in which infected poultry were kept. Recommended PPE to be worn in addition to normal overalls and gumboots included disposable overalls, gloves, protective eyewear, and masks capable of preventing inhalation of aerosolized virus particles. Handwashing with a disinfectant soap after handling of any potential contaminated material was also advocated.^24^ The NICD also advised that persons exposed to infected poultry or their products should be followed up for 7‐10 days to identify influenza symptoms, in which case samples should be collected and submitted to the NICD.

### 2.3 Specimens and isolates from animals

Influenza A(H5N8)‐positive (n = 63) specimens consisted of virus isolates (n = 1) cultured in embryonated hen eggs and either pooled (39/63; 62%) or individual (23/63; 36.5%) cloacal; tracheal or multi organ swabs from infected and uninfected (n = 32) birds (chicken, ostrich, guinea fowl, Egyptian geese, pigeon, blue crane, swan, duck, and geese) collected by the Western Cape Department of Agriculture from farms or other localities within the WCP during August 2017. Several samples represented duplicate or pooled swab samples from the same source; therefore, 40% (25/63) represented unique samples.

### 2.4 Laboratory procedures

#### 2.4.1 Testing of human samples for A(H5N8)

Sample nucleic acid extraction was done using the MagNA Pure 96 automated extraction instrument along with the MagNA Pure 96 Small Volume Total Nucleic Acid kit (Roche). Samples were then assayed using the Centres for Disease Control and Prevention (CDC) influenza A/B typing (FluRUO‐01), and subsequent influenza A subtyping kits (H1pdm09/H3/H5a/H5b/H7) (FluRUO‐09, FluRUO‐08) reaction kits acquired from the International Reagent Resource using manufacturer's instructions.^25^, ^26^

#### 2.4.2 Testing of avian samples for influenza A(H5N8)

Avian specimens were initially screened by the Western Cape Department of Agriculture for avian influenza A(H5N8). Total nucleic acid were extracted from specimens using the QIAcube automated nucleic acid extraction platform using the QIAcube HT kit (Qiagen), and AI virus infection was detected using the VetMAX™‐Gold AI virus detection Kit (Life Technologies). Influenza virus H5 hemagglutinin and N8 neuraminidase subtype identities were determined by real‐time PCR using previously published methods.^27^, ^28^

#### 2.4.3 Real‐time PCR assays for avian influenza diagnostics in humans

Two assays were evaluated for their ability to detect A(H5N8) strains in circulation during the outbreak period: (a) The CDC influenza typing (A/B) (FluRUO‐01) assay combined with assays for H1pdm09 and H3, H5, and H7 (FluRUO‐09 and FluRUO‐08) subtyping (International reagent resource (IRR)), and (b) the commercial FluHunter A(H5N8) kit (Genekam). Assays were conducted as described and according to manufacturer's instructions.^25^, ^26^ The FluHunter A(H5N8) kit detects both the H5 and N8 subtypes, using a single primer‐probe set for each target. Congruence of results between the assays was determined by calculating Cohen's Kappa coefficient.^29^

### 2.5 AI A(H5N8) genome amplification and sequencing

For pandemic preparedness, we assessed if procedures routinely used for human influenza A virus characterization can be used to characterize AI A(H5N8) viruses. Briefly, nucleic acid extracts were treated with RNase‐free DNase I (New England Biolabs) to enrich for RNA at 37°C for 30 minutes followed by heat inactivation after addition of 5 µL of 50 mmol/L EDTA. The eight genomic segments of influenza A viruses were simultaneously amplified using a one‐step RT‐PCR.^30^, ^31^ Briefly, enriched RNA served as template in PCR reaction mixture which combined 0.2 μmol/L of each of the cUni‐12; cUni12G; and cUni‐13 primers with SuperScript III one‐step RT‐PCR system with Platinum *Taq* high‐fidelity DNA polymerase system (Thermo Fisher).^30^, ^31^ The temperature cycling profile parameters were 42°C for 60 minutes, 94°C for 2 minutes, followed by five cycles of (94°C for 30 seconds, 45°C for 30 seconds, and 68°C for 3 minutes), followed by 31 cycles of (94°C for 30 seconds, 57°C for 30 seconds, and 68°C for 3 minutes). PCR products were confirmed on a 1% agarose gel. PCR amplicons were enriched for viral templates by MspJI restriction enzyme (New England Biolabs) digestion at 37°C for 16 hours followed by incubation at 72°C for 20 minutes to inactivate the MspJI enzyme.^32^ Specimens were then processed for next‐generation sequencing on the Illumina MiSeq instrument.

### 2.6 Phylogenetic and sequence analysis

Illumina sequencing library was prepared from amplified PCR products using Nextera XT Sample Preparation kit (Illumina). Briefly, PCR amplicons were tagmented enzymatically (55°C for 5 minutes), barcoded through PCR amplification, purified using AMPure XP beads (Beckman Coulter), and normalized and pooled according to manufacturer's protocol. Samples were multiplexed and sequenced using Illumina Miseq v3 kit with 300‐bp paired‐end reads.

The resultant sequenced reads were analyzed using a reference based mapping approach implemented in CLC Genomics Workbench version 11 (Qiagen). Prior to mapping, reads were trimmed for quality and then mapped against concatenated avian influenza A(H5N8) viral segment sequences (Genbank accesions: MF037848, MF037851, MF037852, MF037854, MF037856, MF037858, MF037859, and MF037860) to extract the consensus sequences and perform the variant‐calling analysis. Seven samples (0561, 0065, 0340, 0274, 0243, 0581, 0336) were also sequenced by the Onderstepoort Veterinary Institute (OVI) reference laboratory, South Africa for comparison.

Variations within segment 1 polymerase PB2 (PB2) gene, segment 2 polymerase PB1 (PB1) and putative PB1‐F2 protein (PB1‐F2) genes, segment 3 polymerase PA (PA) and PA‐X protein (PA‐X) genes, segment 4 hemagglutinin (HA), segment 5 nucleocapsid protein (NP) gene, segment 6 neuraminidase (NA) gene, segment 7 matrix protein 2 (M2) and matrix protein 1 (M1) genes, and segment 8 nuclear export protein (NEP) were detected using the low frequency variant detection tool within the CLC Genomics Workbench version 11 (Qiagen). This model discards variants whose representation in the reads is likely due to sequencing errors or mapping artefacts using a statistical test and subsequently reports on positions where there may be single‐nucleotide variation including insertions, deletions and substitutions. Identified variations were further investigated using CLC Genomic workbench to identify amino acid changes. This analysis included determining the presence of known mammalian‐adaptive mutations in NS1 (P42S), HA (A149V, Q222L, and G224S) and PB2 (L89V; E627K; and D701N).^12^, ^13^, ^22^

Concatenated full‐length influenza genome sequences and NA and HA consensus sequences (GISAID accession numbers KY451418 to KY451452) were aligned with international A(H5N8) reference sequences (downloaded from the GISAID sequence database) using the Multiple Sequence Comparison by Log‐Expectation (MUSCLE) algorithm embedded in BioEdit v7.0.9.1.^33^ Maximum likelihood (ML) phylogenetic trees and quantitative pairwise distance matrices were determined using Mega 6.06.^33^ ML trees were constructed using the GTR‐GAMMA nucleotide substitution model, using 100 replicate bootstrap analyses [20]. Nucleotide sequence similarity searches were conducted using the influenza virus BLASTn function of the influenza virus resource database (https://www.ncbi.nlm.nih.gov/genomes/FLU/Database/nph-select.cgi?go=database). The presence of N‐glycosylation sites on envelope glycoproteins (HA and NA) was determined by NetNGlyc 1.0 server (http://www.cbs.dtu.dk/services/NetNGlyc/) using default settings. HA amino acid numbering excluded the signal peptide sequence while NA amino acid numbering included the signal peptide sequence to conform to the methods of.^34^

### 2.7 Ethics

This study was conducted as part of the NICD outbreak response (M160667) and surveillance (M150855) for emerging zoonotic infections study protocols approved by the University of the Witwatersrand Human research ethics committee. Informed consent was obtained from all participants. Permission to perform study under section 20 of the animal diseases act was attained from the South African Department of Agriculture, Forestry and Fisheries.

## 3 RESULTS

### 3.1 Human surveillance

A total of 74 individuals met the case‐under‐investigation definition for a potential AI infection between June 28, 2017, and November 11, 2017 (Table 1). Of these, 54 (73%) were enrolled through active surveillance at three commercial farms and 21 (27%) were identified through passive surveillance of which 3 (4%) were referred from private and state pathology laboratories. The median age was 34 years (range 17‐59 years), 50 (68%) were male, and all were sampled between 7 and 36 days subsequent to the earliest estimated date of exposure to AI. The majority of individuals reported cough (69%; 51/74) and coryza (62%; 46/74), while less than half reported fever (46%; 34/74). Other symptoms reported included a sore throat (54%; 40/74), conjunctivitis (41%; 30/74) and/or shortness of breath (28%; 21/74). Varying combinations of personal protective equipment (PPE) were worn by the sampled and interviewed individuals at the different establishments (Figure S1). The majority (54%, 40/74) indicated that they wore overalls, boots, masks, gloves, and goggles. The 74 samples identified through human surveillance were collected between 7 and 36 days post‐exposure and tested negative for AI A(H5) viruses. Two samples (3%) tested positive for seasonal human influenza A(H3N2) viruses (Table 1).

**Table 1:** Human cases under investigation for avian A(H5N8) from each establishment, South Africa, 2017

Source of samples	Date of sample collection	Time lag between potential exposure and sampling (days)	Exposed workers n	Exposed workers meeting case definition n (%)	Number testing influenza A positive n (%)
Farm 1 (mpumalanga)	28 June‐19 July	9‐30	120	22 (18.3)	0 (0)
Farm 2 (mpumalanga)	7 July	17	35	7 (20.0)	0 (0)
Farm 3 (gauteng)	11 August	36	46	25 (54.3)	0 (0)
NSPCA (gauteng)	‐	7	n/a	6	0 (0)
Western cape veterinary services	5 Sept‐11 Nov	<10 d	n/a	11	0 (0)
Private laboratory referrals (gauteng)	July	n/a	n/a	2	2^a^ (100)
National health laboratory service referrals (western cape)	12 October	<10 d	n/a	1	0 (0)
Total				74	2

Abbrevaitions: n, number; n/a, source population size not available; NSPCA, National Societies for the Prevention of Cruelty to Animals.

a Tested positive for Influenza A(H3N2) seasonal human influenza virus.

### 3.2 Evaluation of influenza A(H5N8) diagnostic assays

Sixty‐three influenza A(H5N8)‐positive nucleic acid extracts and 32 HPAI‐negative specimens were tested. All 63 AI A(H5N8)‐positive veterinary specimens tested positive for influenza A on the CDC assay; 94% (59/63) tested positive with the CDC influenza A/H5 assay (Table 2). The FluHunter A(H5N8) (Genekam) assay was evaluated on 76% (48/63) of veterinary samples positive for influenza A(H5N8); the H5 target was detected in only 6% (3/48) and the N8 target in 92% (44/48) of specimens (Table 2). All 32 specimens from known AIV‐negative birds were correspondingly negative with both the CDC and Flu Hunter assays (Table 2). The kappa coefficients were 1 and 0.91 for the CDC influenza A and CDC H5 assay, respectively, whereas they were 0.07 and 0.89 for the FluHunter H5 and N8 assays, respectively.

**Table 2:** Assay performance of CDC and flu hunter real‐time PCR influenza A, H5 and N8 diagnostic assays on veterinary samples

Tests	A(H5N8)‐positive samples	Influenza A‐negative samples	Sensitivity (%)	Specificity (%)	PPV (%)	NPV (%)	Kappa coefficient
CDC influenza A	63/63	0/32	100	100	100	100	1
CDC H5 assay	59/63	0/32	93.7	100	100	88.9	0.91
Flu hunter assay H5	3/48	0/32	6.3	100	100	41.5	0.07
Flu hunter assay N8	44/48	0/32	91.7	100	100	88.9	0.89

Abbrevaitions: NPV, negative predictive value; PPV, positive predictive value.

### 3.3 Genome sequencing of influenza A(H5N8) derived from veterinary samples

**Figure 1:**
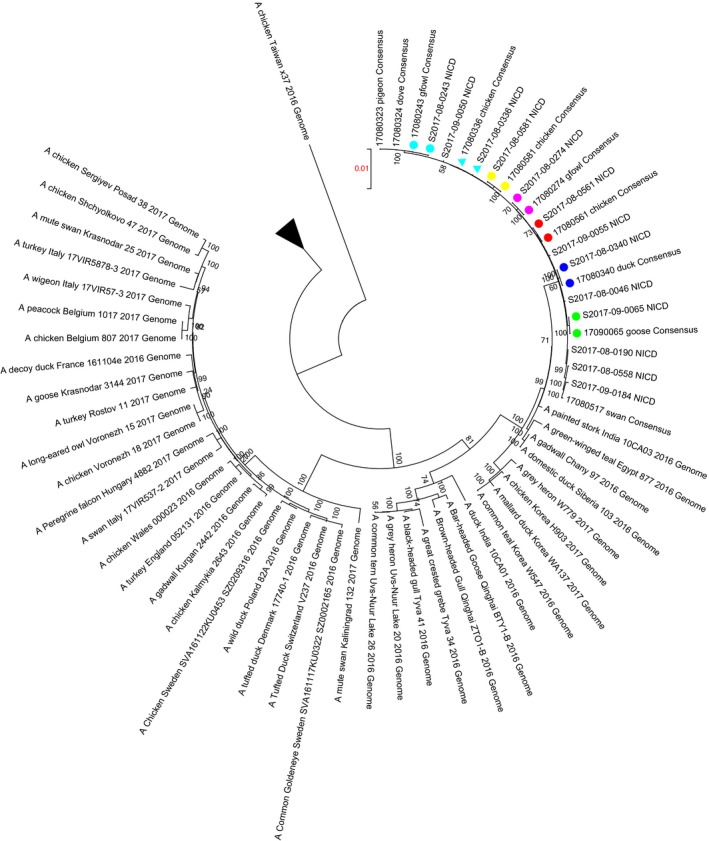
Maximum likelihood tree constructed using Mega 5.0 under GTR‐GAMMA model of nucleotide evolution, displaying phylogeny of concatenated full‐genome WCP A(H5N8) virus sequences (n = 13) in reference to publically available A(H5N8) sequences representative of the global A(H5N8) diversity between 2016 and 2017. Branch with the WCP is indicated by the arrow, and duplicate sequences that formed part of an inter‐laboratory comparison are indicated with correspondingly colored symbols

Influenza A genome amplification and sequencing were done for 58/63 specimens (positive in the IRR‐CDC H5 assay) on the Illumina MiSeq next‐generation sequencing (NGS) platform. NGS genomic coverage varied between samples, yielding complete genomes from 13 specimens, 5‐7 gene segments from 30 and 2 to 4 gene segments from 15 specimens sequenced. As several samples consisted of duplicate or pooled swabs from the same source, 43% (25/58) represented unique samples. When considering these unique samples, we recovered 13/25 (52%) full genomes (eight genomic fragments) and 12/25 (48%) partial genomes (missing the PA, PB1, or PB2 genomic fragments) (Table S1). The accuracy of genome sequence data generated in our laboratory was determined by full‐genome ML tree analysis; comparing with reference sequence data for 7 samples (S2017‐08‐0561_NICD, S2017‐09‐0065_NICD, S2017‐08‐0340_NICD, S2017‐08‐0274_NICD, S2017‐08‐0243_NICD, S2017‐08‐0581_NICD, and S2017‐08‐0336_NICD), which were provided by the OVI reference laboratory(Figure 1). All consensus sequence duplicates generated in our study and by OVI were identical.

AI A(H5N8) sequences from infected birds in the WCP were closely related to group B represented by A(H5N8) sequences derived from Egypt (A/green‐winged teal/Egypt/871/2016) and India (A/painted‐stork/India/10CA03/2016) in 2016, demonstrating average genetic distance distances of 0.0072 (0.007‐0.008) and 0.0058 (0.005‐0.007), respectively. Concatenated full‐genome ML tree analysis shows that all WCP A(H5N8) genomes clustered together with strong bootstrap support (100%) and an overall mean genetic distance across the tree of 0.023 (0.000‐0.078). The monophyletic cluster formed by these sequences demonstrates limited sequence diversity within this geographic region with an overall mean genetic distance of 0.001 (0.000‐0.002).

Consensus sequences demonstrated the absence of HA (A149V, Q222L, and G224S) and PB2 (E627K) mammalian‐adaptive mutations for all virus samples sequenced. Furthermore, our H5N8 consensus sequences all displayed the NS (P42S) and PB2 (L89V) mammalian‐adaptive mutations associated with H5N1 strains. Sequence variant‐calling analysis for each virus sequenced demonstrated homogenous viral populations for adaptation mutations sites in NS (S42), HA (A149, Q222, G226), and PB2 (V89, E627); average read counts were as follows: NS = 33 881 (9329‐57892); HA = 194 (5‐907); and PB2 = 1442 (4‐5493) (Table S2). One viral sample, 0416 contained a heterogeneous viral population in PB2 position 701 where average read counts of 666 (23‐1661) were observed. The dominant viral variant presents at ≈78% of the viral population possessed the wild‐type D‐amino acid, while a minor population of 22% of the viral variants possessed the D701E mutation. The known mammalian‐adaptive D701N mutation was not presented.

## 4 DISCUSSION

Following a widespread outbreak of avian influenza in poultry in South Africa and subsequent surveillance efforts, of the 74 individuals that met our case‐under‐investigation definition, none tested positive for influenza A(H5N8). While two individuals tested positive for seasonal human influenza A(H3N2). Prior to testing of human samples, two influenza A(H5N8) diagnostic assays were evaluated using AI‐positive nucleic acid derived from influenza A(H5N8)‐infected birds from affected farms. The assays demonstrated contrasting capacities at detecting contemporary H5‐positive nucleic acid; we excluded the poorly performing kit for human diagnostics. The absence of H5N8 in humans with occupational exposure suggests a low risk of infection.

As a result of farms being quarantined, an extended lag was observed between exposure of symptomatic individuals and sampling (7‐36 days). It is therefore possible that potential infections by avian influenza may have resolved within this period. The lag time though was estimated from the first date of exposure, which is when the outbreak started on the farm. Subsequent to initial exposure, farm workers also assisted with the euthanasia and disposal of carcasses, which took place over several days. The workers therefore could potentially have been continually exposed throughout the culling operation, which may reduce the estimated lag period between exposure and sampling and limit the possibility of missing resolved infections. In addition to active surveillance conducted at the three commercial poultry farms, surveillance guidelines were also provided to public health clinics within all affected areas. This allowed us to identify any potentially infected individuals through the public health system. Despite these efforts, it is possible that potential human A(H5N8) infection instances could have been missed through failure to report as well as mild or asymptomatic infections, limiting our ability to fully evaluate the risk of human infection.

Varying levels of PPE were used by individuals in direct contact with infected birds at different sites. All sampled individuals wore boots and overalls to prevent exposure, and 54% of them also wore masks, gloves, and goggles. This is in line with approaches used by European Union countries to manage potential human health risks during 2017 A(H5N8) poultry outbreaks, which recommend the use of gloves (21/22 countries), goggles (21/22 countries), masks (21/22 countries), and bodysuits (21/23 countries) for individuals in direct contact with infected birds.^6^

Two influenza diagnostic kits were evaluated for their ability to identify the avian H5 and/or N8 subtypes using AI‐positive and negative nucleic acid extracts derived from infected birds and identified using validated veterinary assays. CDC‐developed influenza PCR assays were selected as they are commonly used assays used by influenza surveillance laboratories, while the Flu Hunter assay was selected because it is specifically designed for the identification of H5N8 infection in humans (contains a human internal control). Kappa coefficients of 0.91, and 0.89 for the CDC H5 and Flu Hunter N8 assays, respectively, demonstrate near perfect agreement with veterinary diagnostic assays. The four known positive samples that the CDC H5 assay failed to detect had high cycle threshold values (>34) for the influenza A typing PCR which may explain failure to amplify due to low template concentration and detection limit of the assay. The Flu Hunter H5 assay Kappa coefficient of <0.1 could be attributed to sequence divergence in the HA PCR target region. The CDC H5 kit was more efficient at identifying contemporary influenza A(H5N8) strains in South Africa as it targets a more conserved region of HA compared to the Flu Hunter assay (unfortunately, the HA target sequence of the Flu Hunter assay was not provided by the manufacturer).

Congruence of sequence data generated by the human and veterinary laboratories indicated the accuracy and suitability of current human influenza protocols for sequencing of avian influenza A(H5) strains. HPAI A(H5N8) sequences from infected birds demonstrated a narrow sequence variance range of 0.000‐0.002 and an overall mean genetic distance of 0.001. When screening for presence of known AI H5N1 and H5N8 mammalian‐adaptive mutations, HA A149V, Q222L, and G224S mutations which enable mammalian receptor binding and PB2 (E627K) adaptive mutations were not presented in any of the consensus sequences (dominant viral species) or minority virus species (homogenous viral population at these positions) in each of the virus samples sequenced.^34^ The PB2 D701N adaptive mutation was previously observed to enable mammalian infection through the substitution of an electrically charged amino acid (D) for a neutral amino acid chain (N). While this mutation was not observed in any samples, sample 0416 sequenced in this study had a minority viral population (22%) that possessed the D701E mutation. All WCP A(H5N8) samples sequenced possessed the NS (P42S) and PB2 (L89V) mutations noted as markers of mammalian adaptation.^12^, ^13^, ^15^, ^16^, ^22^ However, the presence of these adaptation markers alone may not be sufficient to enable adaptation as no human infections with influenza A(H5N8) strains were detected or reported in South Africa or elsewhere.

Our surveillance activities have found no human H5N8 infection, and sequence analyses have also shown no clear impression of influenza A(H5N8) adaptation for mammalian infection. Due to insufficient sampling and sampling lag limitations, we are unable to confidently evaluate the true risk of influenza A(H5N8) transmission to humans. It is recommended that a follow‐up study looking at influenza A(H5N8) sero‐positivity of individuals not sampled in this study is conducted. However, combined with a wealth of data from global A(H5N8) surveillance studies, our results corroborate findings suggesting that A(H5N8) in its current form is a low‐risk human pathogen.^6^ Furthermore, to evaluate the effect of observed adaptive mutations, follow‐up cell culture‐based or animal infection model studies need to be conducted. Due to the high mutation rate of RNA viruses and the high frequency of AI outbreaks, constant surveillance at the human‐animal interfaces is important for the control or aversion of potential zoonotic and novel pandemic events.
