# Supplementary material for: Human surveillance and phylogeny of highly pathogenic avian influenza A(H5N8) during an outbreak in poultry in South Africa, 2017
Source: Influenza Other Respir Viruses. 2020 Feb 14;14(3):266–73. doi: 10.1111/irv.12724 (PMC7182598; doi:10.1111/irv.12724)
Supplement: Supplementary file 1 [file IRV-14-266-s001.docx]

**Supporting information:**

**Supporting Information Figure 1:** Combinations of personal protective equipment (PPE) reported worn for individuals in direct contact with A(H5N8)-infected birds.

**Supporting Information Table 1.** Genome fragments sequenced for 2017 South African influenza A(H5N8) for unique viral samples. Red cell shading indicates that the genome fragment was sequenced.

| **Sample ID** | **PB2** | **PB1** | **PA** | **HA** | **NP** | **NA** | **MP** | **NS** | **Complete/partial genome** |
| --- | --- | --- | --- | --- | --- | --- | --- | --- | --- |
| **0046-AF** |  |  |  |  |  |  |  |  | Complete |
| **0050-56** |  |  |  |  |  |  |  |  | Complete |
| **0055-P2** |  |  |  |  |  |  |  |  | Complete |
| **0065-P2** |  |  |  |  |  |  |  |  | Complete |
| **0184-63** |  |  |  |  |  |  |  |  | Complete |
| **0558-P2** |  |  |  |  |  |  |  |  | Complete |
| **0561-P1** |  |  |  |  |  |  |  |  | Complete |
| **0581-P1** |  |  |  |  |  |  |  |  | Complete |
| **0190-9** |  |  |  |  |  |  |  |  | Complete |
| **0243-P1** |  |  |  |  |  |  |  |  | Complete |
| **0274-P1** |  |  |  |  |  |  |  |  | Complete |
| **0336-P1** |  |  |  |  |  |  |  |  | Complete |
| **0340-P1** |  |  |  |  |  |  |  |  | Complete |
| **0047-P7** |  |  |  |  |  |  |  |  | Partial |
| **0161-P8** |  |  |  |  |  |  |  |  | Partial |
| **0268-P2** |  |  |  |  |  |  |  |  | Partial |
| **0275-P1** |  |  |  |  |  |  |  |  | Partial |
| **0323-P1** |  |  |  |  |  |  |  |  | Partial |
| **0324-24** |  |  |  |  |  |  |  |  | Partial |
| **0361-P17** |  |  |  |  |  |  |  |  | Partial |
| **0362-P8-33** |  |  |  |  |  |  |  |  | Partial |
| **0416-38** |  |  |  |  |  |  |  |  | Partial |
| **0481-P2** |  |  |  |  |  |  |  |  | Partial |
| **0517-P2** |  |  |  |  |  |  |  |  | Partial |
| **0520-46** |  |  |  |  |  |  |  |  | Partial |

**Supporting Information Table 2.** Next generation sequencing genomic coverage, average read counts and range for genomic positions where mammalian adaptive mutations have been identified.

| Genomic fragment | NS | HA | | | PB2 | |
| --- | --- | --- | --- | --- | --- | --- |
| Amino acid position | S42 | A149 | Q222 | G226 | V89 | E627 |
| Coverage | 33881 (9329-57892) | 200 (5-907) | 189 (6-859) | 192 (6-868) | 1442 (4-5493) | 1442 (4-5493) |
